# A simple high-performance matrix-free biomass molten carbonate fuel cell without CO_2_ recirculation

**DOI:** 10.1126/sciadv.1600772

**Published:** 2016-08-17

**Authors:** Rong Lan, Shanwen Tao

**Affiliations:** 1 School of Engineering, University of Warwick, Coventry CV4 7AL, UK.; 2 Department of Chemical Engineering, Monash University, Clayton, Melbourne, Victoria 3800, Australia.

**Keywords:** Biomass, molten carbonate fuel cell, CO2 recirculation, carbon

## Abstract

In previous reports, flowing CO_2_ at the cathode is essential for either conventional molten carbonate fuel cells (MCFCs) based on molten carbonate/LiAlO_2_ electrolytes or matrix-free MCFCs. For the first time, we demonstrate a high-performance matrix-free MCFC without CO_2_ recirculation. At 800°C, power densities of 430 and 410 mW/cm^2^ are achieved when biomass—bamboo charcoal and wood, respectively–is used as fuel. At 600°C, a stable performance is observed during the measured 90 hours after the initial degradation. In this MCFC, CO_2_ is produced at the anode when carbon-containing fuels are used. The produced CO_2_ then dissolves and diffuses to the cathode to react with oxygen in open air, forming the required ${\text{CO}}_{3}^{2-}$ or ${\text{CO}}_{4}^{2-}$ ions for continuous operation. The dissolved $O_{2}^{-}$ ions may also take part in the cell reactions. This provides a simple new fuel cell technology to directly convert carbon-containing fuels such as carbon and biomass into electricity with high efficiency.

## Teaser

A matrix-free molten carbonate fuel cell without CO_2_ recirculation for efficient power generation from carbon-containing fuels.

## INTRODUCTION

In terms of energy sources, about 87% of the energy consumed in the world depends on fossil fuels (*1*), which are a major source of CO_2_ emission. It is desirable to integrate more renewable energy sources with lower carbon footprints. Biomass from energy crops, agricultural residues, wastes and residues, and forestry is an important renewable energy source. It is estimated that global biomass potential is about 200 EJ (up to 600 EJ), which is about one-third of the world’s total energy consumption (*2*). Biomass energy is used to generate heat through combustion or to generate electricity by supplying steam for the same kind of steam-electric generators used to burn fossil fuels. Fuel cell technology is another type of technology that is used to convert the chemical energy in biomass into electricity. Biomass can be used to produce charcoal through pyrolysis. The charcoal produced is an ideal fuel for direct carbon fuel cells (DCFCs) (*3*–*5*). Alternatively, biomass, such as starch, cellulose, and sugar, can be used as fuel for electricity generation through microbial fuel cells, but the power density is quite small (Table 1) (*6*–*8*). It has been determined that intermediate- to high-temperature fuel cells, such as molten alkaline fuel cells, molten carbonate fuel cells (MCFCs), solid oxide fuel cells (SOFCs), and hybrid MCFC/SOFC, use carbon or charcoal derived from biomass for power generation with reasonably high power density (*3*–*5*, *9*–*14*). Liquid tin anode SOFCs were also investigated for DCFCs (*15*), but the deposition of nonconductive SnO_2_ at the anode triple-phase boundary is a potential problem (*3*). The high requirements of materials in contact with the liquid anode are also potential challenges. Lignin was also reported as fuel for a hybrid MCFC/SOFC fuel cell with a power density of 25 mW/cm^2^ at 560°C (*16*). An unprecedented high power density of 878 mW/cm^2^ has been achieved at 750°C when a hybrid MCFC/SOFC was used for DCFCs using a special charcoal (a pyrolyzed medium-density fiberboard) as the fuel (*5*). However, conventional SOFC technology does not yet meet the reliability and durability targets for large-scale commercialization. Although materials improvement plays a central role in the development process, system and stack design must integrate degradation analysis to achieve the lowest cost of electricity (*17*). The introduction of molten carbonate in the hybrid MCFC/SOFC DCFCs will make the hybrid MCFC/SOFC technology more challenging, particularly on materials in contact with the molten carbonate. Therefore, it is necessary to develop DCFCs or direct biomass fuel cells based on a simple design to avoid the challenges associated with the complicated cell design.

**Table 1:** Comparison of reported typical fuel cell performances on using carbon and biomass as the fuel. YSZ, yttria-stabilized zirconia.

**Electrolyte**	**Charge** **carrier**	**Cathode**	**Anode**	**Fuel**	**Oxidant**	**Temperature** **(°C)**	**OCV** **(V)**	**Power** **density** **(mW/cm)**	**References**
NaOH/KOH (54:46 molar ratio)	OH^−^	Nickel wound tube	Nickel mesh	Activated carbon	O_2_/air + H_2_O	500	~0.91	~38.5	(*10*)
YSZ	O^2−^	La_0.8_Sr_0.2_MnO_3_ + Ce_0.9_Gd_0.1_O_2-δ_	(Ni_0.9_-Fe_0.1_)- Ce_0.9_Gd_0.1_O_2-δ_	Charcoal	O_2_	800	1.0	35	(*9*)
YSZ	O^2−^	La_0.6_Sr_0.4_CoO_3-δ_	Ni-YSZ	Mixture of pyrolyzed fiberboard and Li_2_CO_3_-K_2_CO_3_ (62:38 mol)	Flowing air	750	1.013	878	(*5*)
38% Li_2_CO_3_ + 62% K_2_CO_3_	CO_3_^2−^	LiNiOx on stain less steel	Ni-coated stainless steel	Soot	Flowing CO_2_ + O_2_ (molar ratio, 1:1)	800	~1.04	~96	(*24*)
32 wt % Li_2_CO_3_ + 68 wt % K_2_CO_3_	CO_3_^2−^	Ag sheet	Porous Ni rod	Graphite	Flowing CO_2_ + O_2_ (molar ratio, 2:1)	700	~1.0	~640	(*25*)
Molten (Li,Na,K)_2_CO_3_	CO_3_^2−^(O_2_^−^)	Ag	Ag	Bamboo charcoal	Static air	800	0.97	430	This study
Nafion 212	H^+^	Pt/C	Carbon paper/ carbon nanotube/ enzyme	0.01 mM maltodextrin	Air	50	~0.6	0.8	(*7*)
Nafion 117	H^+^	Pt/C	Pt/C	Starch processing waste water	Air	30	0.49	0.0239	(*38*)
Nafion 117	H^+^	Pt/C	Carbon cloth (C)	Lignin in H_3_PMo_12_O_40_ solution	Flowing O_2_	Room temperature	~0.37	~0.55	(*6*)
Ce_0.8_Sm_0.2_O_2−δ_^−^ (Li,Na)_2_CO_3_	O^2−^/CO_3_^2−^	Lignin + active carbon Ce_0.8_Sm_0.2_O_2−δ_- (Li,Na)_2_CO_3_	Composite Li/Cu/Ni/Zn oxides	Lignin	Flowing air	560	~0.74	25	(*16*)
62 mol % Li_2_CO_3_ + 38 mol % K_2_CO_3_	CO_3_^2−^	Ag	Ag	62 mol % Li_2_CO_3_ + 38 mol % K_2_CO_3_ + 5 wt % carbon	Flowing CO_2_ + air	700	0.9	34	(*11*)
Molten (Li,Na,K)_2_CO_3_	CO_3_^2−^(O_2_^−^)	Ag	Ag	Wood	Static air	800	0.9	410	This study

MCFC is an important type of fuel cell for efficient power generation (*18*, *19*). Although significant progress has been made in the last 40 years, MCFC technology still faces challenges, such as low power density, the stability of LiAlO_2_ matrix in the carbonate/LiAlO_2_ electrolyte (*20*), and the corrosion of electrode and bipolar materials (*21*, *22*). There is also a requirement to recirculate CO_2_ from the anode exhaust to the cathode to react with O_2_ in air to generate ${\text{CO}}_{3}^{2-}$ ions as the charge carriers according to reaction 1 and to maintain the carbonate composition. This also complicates the balance-of-plant equipment (*18*)

CO2+12O2+2e−→CO32− (1)

Recirculation of CO_2_ in MCFC not only complicates the system but also has a significant negative effect on overall efficiency (*23*). The use of a matrix also increases the internal resistance, leading to reduced power density. Therefore, matrix-free MCFCs give a higher power density.

MCFCs are attractive for DCFCs/direct biomass fuel cells because of their excellent wetting property to the solid fuels, which achieve a high power density, and their high tolerance to impurities (*3*). Ahn *et al.* (*11*) reported a direct carbon MCFC (DCMCFC), where carbon fuel [5 weight percent (wt %)] was premixed with a molten carbonate electrolyte, with air and CO_2_ bubbled through a porous cathode tube. In another report, a porous anode tube was used for a DCMCFC (*24*). In both cells, flowing CO_2_ at the cathode is essential (*11*, *24*). Air bubbling through the cathode is in direct contact with the fuel and, thus, chemically reacts with the carbon fuel, forming CO or CO_2_. This direct reaction will generate heat rather than electricity, leading to decreased electrical efficiency (*3*). In a recent report where a porous nickel rod was used as the anode, mixed graphite and (Li,K)_2_CO_3_ was used as slurry fuel, and mixed CO_2_ and O_2_ was flowed at the cathode (*25*), good performance was achieved.

**Fig. 1:**
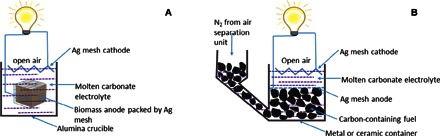
Schematic diagram of a matrix-free MCFC without flowing CO_2_ at the cathode. (**A**) Caged anode. (**B**) Design for continuous operation.

Here, we report a simple design for MCFCs that does not involve flowing CO_2_ at the cathode (Fig. 1A). In this design, solid fuel is put in a cage made of conducting materials such as silver mesh, eliminating the need to flow CO_2_ at the cathode. This will greatly simplify the cell manufacturing process and reduce the operating cost. Continuous fuel cell operation can be achieved through a cell design shown in Fig. 1B. Solid fuels such as carbon or biomass are ideal for this new type of MCFC.

In our new design, unlike previously reported MCFCs (*11*, *24*), air or oxygen was not bubbling into the molten carbonate electrolyte. The only possible direct chemical reaction between the fuel and oxygen is through dissolved molecular oxygen (O_2_). It has been reported that, at a temperature of 600°C, the dissolved oxygen in 62 mole percent (mol %) Li_2_CO_3_–38 mol % K_2_CO_3_ and that in 52 mol % Li_2_CO_3_–48 mol % Na_2_CO_3_ are in charged forms, such as superoxide ($O_{2}^{-}$) or peroxymonocarbonate (${\text{CO}}_{4}^{2-}$), and the dissolved molecular oxygen (O_2_) at high temperatures is negligible (*26*). In the reaction between charged oxygen species and the anode, there must be a loss of electrons to generate electricity. Under this circumstance, the charged dissolved oxygen is used as the charge carrier. The possible cathode reactions are

$$O_{2}+e^{-}\to O_{2}^{-}$$ (2)

$${\text{CO}}_{2}+O_{2}+2e^{-}\to {\text{CO}}_{4}^{2-}$$ (3)

The formed anions will diffuse in the molten carbonate and react with the solid fuel at the anode. If carbon is used as the fuel, the reactions at the anode are

$$C+O_{2}^{-}\to {\text{CO}}_{2}+e^{-}$$ (4)

$$C+{\text{CO}}_{4}^{2-}\to 2{\text{CO}}_{2}+2e^{-}$$ (5)

$$C+2{\text{CO}}_{3}^{2-}\to 3{\text{CO}}_{2}+4e^{-}$$ (6)

For reactions 2 and 4, the charge carriers are $O_{2}^{-}$ ions; thus, CO_2_ is not required for the reaction. Therefore, theoretically, an MCFC can work without flowing CO_2_.

In our new cell design, at high working temperatures, the efficiency loss due to the direct reaction between molecular oxygen and fuelwill be minimized because of the low solubility of molecular O_2_ in molten carbonates (*26*). Moreover, the possible stability issues associated withdecomposition of carbonates can also be avoided because of the in situ formation of CO_2_ at the anode, which suppresses carbonate decomposition. CO_2_ solubility in molten carbonates is much higher than that of O_2_ (*24*, *26*, *27*). The solubility of CO_2_ in the molten ternary eutectic mixture Li_2_CO_3_-Na_2_CO_3_-K_2_CO_3_ (43.5/31.5/25.0 mol %) at 700°C was 9.5 × 10^−2^ mol/liter (*27*). Thus, the produced CO_2_ at the MCFC anode can dissolve and diffuse to the cathode through the molten carbonates to react with O_2_ in air, forming ${\text{CO}}_{4}^{2-}$ and ${\text{CO}}_{3}^{2-}$ ions according to reactions 1 and 3. The formed ${\text{CO}}_{4}^{2-}$ and ${\text{CO}}_{3}^{2-}$ ions then diffuse to the anode to react with the fuel according to reactions 5 and 6. Under this circumstance, flowing CO_2_ at the cathode is not required in the matrix-free MCFCs. If the CO_2_ generated at the anode is beyond the solubility limit in molten carbonates, the “extra” CO_2_ may also pass through the electrolyte to the cathode and then react with O_2_ in air, forming carbonate ions to take part in the fuel cell reaction.

## RESULTS

### Direct charcoal MCFC

On the basis of the analysis above, two fuel cells were tested using thenewly designed cell shown in Fig. 1A, one with the anode made of bamboo-derived charcoal and the other made of wood. Biomass was used as fuel in the fuel cell because it is nearly carbon-neutral. The mixture of Li_2_CO_3_, Na_2_CO_3_, and K_2_CO_3_ was used as the electrolyte, and the silver mesh cathode was exposed to static air without CO_2_ flow. Experimental details can be found in the Supplementary Materials.

**Fig. 2:**
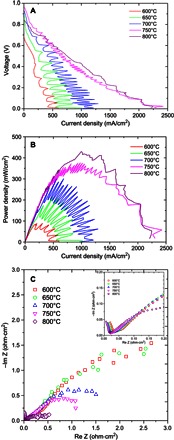
(**A** to **C**) *I*-*V* curves (A), *I*-*P* curves (B), and ac impedance spectra (C) of DCMCFC.

The fuel cell performance of the direct charcoal fuel cell is shown in Fig. 2A. At 600°C, the open-circuit voltage (OCV) of the cell was 0.69 V. Maximum current and power densities of ~500 mA/cm^2^ and 88 mW/cm^2^, respectively, were observed. A key feature on the *I*-*V* curve (Fig. 2A) is the oscillation of the curve at high current densities. This is due to the formation of gas (CO and/or CO_2_) on the charcoal surface, which may temporarily separate the contact between the silver anode and the charcoal, leading to a lower current. However, once the gas bubble is released or dissolved in the molten carbonates, the anode/charcoal contact is recovered and a large current density is observed. The formed CO may be oxidized in situ to CO_2_ by the anions, such as $O_{2}^{-}$, ${\text{CO}}_{3}^{2-}$, and ${\text{CO}}_{4}^{2-}$. When the operating temperature increased to 650°C, the OCV jumped to 0.9 V. This is fairly close to the theoretical OCV of DCFCs (1.0 V) (*3*). At 750°C, the OCV was 0.98 V, and then it decreased to 0.85 V when the operating temperature further increased to 800°C. The OCV drop may be related to the formation of intermediate CO at the anode. At high temperatures, CO might be formed via the reverse Boudouard reaction

$$C+{\text{CO}}_{2}\to 2\text{CO}$$ (7)

This reaction is spontaneous at a temperature above 700°C (*28*). The presence of alkali carbonates, such as Na_2_CO_3_ and K_2_CO_3_, will suppress the formation of CO; therefore, decreased OCV was shifted to 800°C in our study (*29*). The generated CO can be electrochemically oxidized in situ to CO_2_ according to the reaction

$$\text{CO}+{\text{CO}}_{3}^{2-}\to 2{\text{CO}}_{2}+2e^{-}$$ (8)

whereas the OCV for oxidation of CO to CO_2_ decreased at elevated temperatures (*3*, *30*, *31*). Decreased OCV at high temperatures was also observed for DCFC based on ceria-gadolinia electrolyte (*31*). When alkali carbonate was used as electrolyte, interactions involving the alkali metal in the melt may have contributed to the observed cell potential, which is complicated (*3*, *28*). At 800°C, the maximum current and power densities were 2.2 A/cm^2^ (Fig. 2A) and 430 mW/cm^2^ (Fig. 2B). This power density is slightly lower than that of the highest DCFC based on the hybrid MCFC/SOFC electrolyte (*5*) and MCFC when mixed CO_2_ and O_2_ was flowing at the cathode (*25*), but is higher than the power density of DCFCs reported by other groups (Table 1). However, the cell design in this study is much simpler than the reported hybrid MCFC/SOFC (*5*) cell and the MCFC (*25*), thus making it easier to fabricate and maintain. Moreover, this is just a starting point, and it is expected that more efficient electrode materials can be identified to further improve the power density. The impedance spectra measured at the OCV of the direct charcoal fuel cell are shown in Fig. 2C. At 800°C, the overall resistance was 0.53 ohm·cm^2^. From the slope of the *I*-*V* curve, it was found that the resistance of the cell decreased at a lower voltage and at a higher current density (Fig. 2A). At a high current density, a large amount of CO_2_ is produced. The produced CO_2_ will increase the local CO_2_ concentration in the melts and cathode and, thus, increase the ${\text{CO}}_{3}^{2-}$ ion concentration, resulting in a higher current density. However, in other DCFCs, a current drop at low operating voltage was observed because of the short supply of fuel (*5*, *11*). This was not observed in this study because of the good contact between the silver anode and the charcoal fuel. The current oscillation at 800°C was not significant, possibly because of the increased solubility of CO_2_ at higher temperatures. The OCVs of the direct charcoal MCFC at different temperatures are shown in fig. S1. The highest OCV of 0.98 V was observed at 750°C. This is slightly lower than the theoretical OCV of DCFCs (~1.0 V). In a DCFC, partial oxidation of carbon to CO can lead to a higher OCV (>1.0 V) (*3*, *5*, *28*).

C+12O2→CO (9)

The fact that the OCV of the cell is slightly lower than 1.0 V indirectly indicates that the complete oxidation of carbon to CO_2_ is the dominant reaction, implying high electricity efficiency (*3*).

**Fig. 3:**
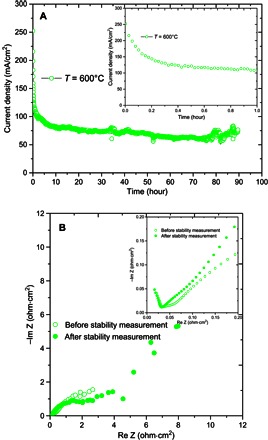
(**A** and **B**) Stability of DCMCFCs at 600°C at an operating voltage of 0.5 V (A) and the ac impedance spectra before and after the stability measurement at 600°C (B).

The performance stability of the DCMCFC at 600°C is shown in Fig. 3A. The operating voltage was 0.5 V. Some initial decrease in current density was observed in the first 20 min, and then the cell tended to give a stable performance. This decrease could be related to the loss of contact between the charcoal and the silver mesh due to the consumption of carbon on the surface during the fuel cell measurement. This may lead to increased electrode polarization resistance that is confirmed by ac impedance spectra for the cell before and after the stability measurements (Fig. 3B). It is expected that better contact with the anode can be achieved for the design shown in Fig. 1B.

Scanning electron microscopy (SEM) observations were carried out on the remaining charcoal after fuel cell measurements as well as before the measurements (fig. S1, A and B). It can be seen that the charcoal is quite porous with close pores. This type of microstructure is not ideal as compared to microstructures with open pores, which allow the diffusion of carbonate melt into the pore, thus extending the anode reaction zone and decreasing the anode polarization resistance, leading to high power density (*32*). It is believed that the power density of DCFCs based on molten carbonate electrolytes can be further improved if carbon with open pores is used as the fuel. Microstructure change of the remaining charcoal during the measurement was insignificant, as shown in fig. S1B. Energy dispersive spectroscopy (EDS) of the charcoal before and after fuel cell measurements is shown in fig. S2. It can be seen that the major composition of the charcoal is carbon, which is expected. After the fuel cell measurement and removal of the electrolyte by water, besides carbon, a tiny amount of magnesium (about 1 wt %) appears in the EDS pattern. The possible source of the magnesium is the internal part of the charcoal, which was exposed to the surface after the surface charcoal was consumed during the fuel cell operation. Another possible source is the carbonate electrolyte; MgCO_3_ is insoluble in water, and thus sticks to the charcoal surface. The signal for oxygen is from the air in the SEM chamber, although there could be oxygen in wood.

### Direct wood MCFC

Because charcoal is normally produced through pyrolysis of biomass such as wood, bamboo, starch, and sawdust, during this pyrolysis, tar, bio-oil, and biogas are formed along with the charcoal. These are also potential fuels for MCFCs for power generation (*33*). The combustion of this produced biogas in the air can provide heat to maintain the high operating temperature. Therefore, directly using biomass such as wood as the fuel in an MCFC can maximize energy utilization. Therefore, wood, which is a typical biomass, was directly used as fuel for the newly designed MCFC (Fig. 1A).

**Fig. 4:**
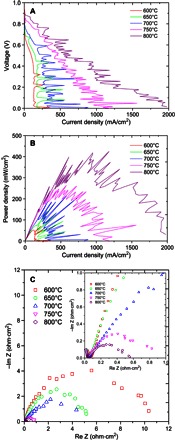
(**A** to **C**) *I*-*V* curves (A), *I*-*P* curves (B), and ac impedance spectra (C) of direct wood MCFC.

When wood was used as the fuel, it was partially pyrolyzed during the heating-up process for the cell. At 600°C, an OCV of 0.96 V was observed. The maximum current and power densities were 150 mA/cm^2^ and 52 mW/cm^2^, respectively. Oscillation of the *I*-*V* curve was observed at all measured temperatures. This is probably related to the in situ pyrolysis of the wood that may produce biogas bubbles, thus temporarily separating the contact of the fuel and silver anode. Higher fuel cell performance was observed at elevated temperatures. At 800°C, the observed OCV was 0.90 V. The OCV of wood MCFC is fairly close to that of direct charcoal MCFC (fig. S3). EDS analysis indicates that a small amount of Si [likely in the form of SiO_2_ or M_2_SiO_3_ (M = Li, Na, or K) as described below] in wood might slightly affect the catalytic activity of the anode; thus, the OCV needs further investigation, although SiO_2_ may be converted into M_2_SiO_3_ (M = Li, Na, or K). Maximum current and power densities of 1.98 A/cm^2^ (Fig. 4A) and 410 mW/cm^2^ (Fig. 4B), respectively, were achieved at 800°C. This power density is just slightly lower than that of direct charcoal MCFC at the same temperature (430 mW/cm^2^). The application of direct biomass MCFCs will save additional costs on a pyrolysis reactor, and the in situ formed by-products—tar, bio-oil, and biogas—are directly converted into electricity or combustion to heat up the fuel cell. This is a better choice for biomass-to-electricity conversion. At 800°C, the overall resistance of the wood MCFC was 0.56 ohm·cm^2^ (Fig. 4C). Again, the measured resistance at OCV could be slightly higher because the initial activation of carbon or biomass could be difficult, particularly at lower temperatures (*11*). Figure S2 (C and D) shows the SEM pictures of the wood before and after fuel cell measurement. The wood is dense before fuel cell measurement, whereas a porous cracked charcoal was obtained after the fuel cell measurement, indicating the occurrence of in situ pyrolysis. EDS analysis indicates that the wood is composed of C, N, and O with a tiny amount of Si, whereas H cannot be picked up by EDS because it is too light (fig. S2). After the fuel cell measurement, the wood was converted into charcoal, and carbon and a small amount of Si (~0.2 wt %) were observed. It is believed that, after fuel cell measurement, Si is likely presented in the form of SiO_2_ or further reacts with molten carbonate electrolyte to form M_2_SiO_3_ (M = Li, Na, or K). The SiO_2_ in biomass is a typical poisoner for the anode for SOFCs (*32*, *34*, *35*). However, in our fuel cell design, the biomass fuel is merged in molten carbonate electrolyte. The SiO_2_ will react with alkali carbonate to form M_2_SiO_3_ (M = Li, Na, or K).

$$M_{2}{\text{CO}}_{3}+{\text{SiO}}_{2}\to M_{2}{\text{SiO}}_{3}+{\text{CO}}_{2}$$ (10)

The formed M_2_SiO_3_ (M = Li, Na, or K) will precipitate on the bottom to be removed later. It has been reported that the reaction between Li_2_CO_3_ and SiO_2_ starts at a temperature around 600°C (*36*). From this point of view, MCFC has better chemical compatibility with silicon than SOFCs.

To determine the residual of the charcoal and wood after being used as fuels in an MCFC, thermogravimetric–differential scanning calorimetry (TG-DSC) analysis was carried out in air (fig. S4). It was found that the solid residual was only 1.47 and 0.90 wt % for charcoal and wood, respectively. Exothermal peaks accompanying weight loss are due to the combustion of charcoal or wood (fig. S4). As mentioned previously, the residual may or may not react with molten carbonate electrolyte. For the MCFC stack described in Fig. 1, after operating for a long period of time, the solid impurities can be separated from the alkali carbonate by dissolving the electrolyte in water because alkali carbonates are water-soluble. After evaporating the water in the carbonate solution, the carbonates are recovered to be used as electrolyte for MCFC again. From this point of view, MCFC has a high impurity tolerant limit, and the contamination is regenerative, which is particularly suitable for fuels containing high impurity, such as biomass.

## DISCUSSION

In previous reports, flowing CO_2_ at the cathode is essential for either the conventional MCFCs (*37*) based on molten carbonate/LiAlO_2_ electrolyte or the new matrix-free MCFCs (*11*, *24*, *25*). Here, for the first time, we demonstrated that flowing CO_2_ is not required for a newly designed MCFC (Fig. 1). In this new design, CO_2_ is produced at the anode when a carbon-containing fuel was used with this CO_2_ dissolving in the molten salt electrolyte and then diffusing to the cathode to react with oxygen in air, forming the required ${\text{CO}}_{3}^{2-}$ ions for continuous operation of the MCFC. Under the fuel cell operating conditions, involvement of other anions such as $O_{2}^{-}$ and ${\text{CO}}_{4}^{2-}$ cannot be ruled out. The presence of CO_2_ at the cathode is not required when $O_{2}^{-}$ ions play the role of charge carriers. From this point of view, recirculation of CO_2_ to the cathode is not required for MCFCs if a carbon-containing fuel is used. Here, high power densities of 430 mW/cm^2^ (4.3 kW/m^2^) and 410 mW/cm^2^ (4.1 kW/m^2^) were achieved when charcoal and wood, respectively, were used as fuel. This newly designed MCFC can be used by coal- or biomass-fired power stations for power generation via direct biomass-to-electricity conversion. We used silver mesh as both cathode and anode, but low-cost electrode materials could be identified to further reduce the cost of the cell. One option is to coat a thin layer of silver on the surface of the electrodes. The application of this type of fuel cell can be further extended to other carbon-containing fuels such as coal and food waste. The application of this novel-type MCFC will markedly increase the conversion efficiency from chemical energy to electricity while also reducing CO_2_ emissions.

## MATERIALS AND METHODS

### Fabrication of the fuel cells

The ternary eutectic salt [(Li,Na,K)_2_CO_3_] was prepared by a solid-state reaction. Lithium carbonate (Li_2_CO_3_; 98%, Alfa Aesar), sodium carbonate (Na_2_CO_3_; 99.5+%, Aldrich), and potassium carbonate (K_2_CO_3_; 99%, Alfa Aesar) were mixed with a molar ratio of 43.5:31.5:25. The mixture was ball-milled in isopropanol for 2 hours using a Pulverisette 6 Fritsch miller at a speed of 400 rpm and dried on a hot plate. The bamboo charcoal (bought from China) or wood (obtained from a local shop) fuel was put in a cage made of silver mesh (Alfa Aesar). The volume of the charcoal or wood was around 1 cm^3^. This caged anode was put in an alumina crucible, and the ternary (Li,Na,K)_2_CO_3_ salts were added in the crucible. The cathode was also made of silver mesh but was folded into a zigzag shape (Fig. 1) to increase the cathode surface area in contact with the ternary (Li,Na,K)_2_CO_3_ salts. Silver wires were mounted on the cathode and anode of the cell. The whole cell was put in a muffle furnace, heated up to 450°C, and dwelled for 1 hour to melt the carbonates. The gap between the anode and the cathode was 10 mm. The cathode area in contact with the molten carbonate was 0.2 cm^2^ for the charcoal fuel cell and 0.4 cm^2^ for the wood fuel cell. The anode area was larger than that for the cathode. The cathode area was taken as an effective area of the cell for current and power density calculation. A K-type thermal couple was mounted near the cell to measure the real temperature of the cell.

### Fuel cell measurements

The as-prepared cell was mounted on an alumina tube with a ceramic adhesive. The cell was put in the middle of a split vertical furnace. A Solartron 1470E electrochemical interface with an integrated Solartron 1455A ac impedance analyzer was used for *I*-*V* and impedance measurements, respectively. The impedance spectra were recorded at an OCV with an applied bias of 100 mV in the frequency range of 1 MHz to 0.01 Hz.

### SEM observation

SEM observation was carried out on a Hitachi SU6600 Field-Emission SEM (FE-SEM). The FE-SEM is equipped with EDS Oxford Inca 350 with a 20-mm X-Max detector.

### Thermal analysis

TG-DSC analyses of charcoal and wood were carried out on a NETZSCH F3 thermal analyzer in flowing air at 900°C with an airflow rate of 50 ml/min.

## Supplementary Material

http://advances.sciencemag.org/cgi/content/full/2/8/e1600772/DC1

## SUPPLEMENTARY MATERIALS

Supplementary material for this article is available at http://advances.sciencemag.org/cgi/content/full/2/8/e1600772/DC1 fig. S1. The SEM pictures of charcoal and wood. fig. S2. EDS spectra of charcoal and wood. fig. S3. The OCV of the charcoal (□) and wood (○) fuel cell. fig. S4. TG-DSC analyses of charcoal and wood.
