# Supplementary material for: A simple high-performance matrix-free biomass molten carbonate fuel cell without CO2 recirculation
Source: Sci Adv. 2016 Aug 17;2(8):e1600772. doi: 10.1126/sciadv.1600772 (PMC4988772; doi:10.1126/sciadv.1600772)
Supplement: http://advances.sciencemag.org/cgi/content/full/2/8/e1600772/DC1 [file supp_2_8_e1600772__index.html]

Science Advances | Science Advances

## Supplementary Materials

**This PDF file includes:**

- fig. S1. The SEM pictures of charcoal and wood.
- fig. S2. EDS spectra of charcoal and wood.
- fig. S3. The OCV of the charcoal (□) and wood (○) fuel cell.
- fig. S4. TG-DSC analyses of charcoal and wood.

Download PDF

**Files in this Data Supplement:**

- Adobe PDF - 1600772\_SM.pdf
