# Supplementary material for: A simple high-performance matrix-free biomass molten carbonate fuel cell without CO2 recirculation
Source: Sci Adv. 2016 Aug 17;2(8):e1600772. doi: 10.1126/sciadv.1600772 (PMC4988772; doi:10.1126/sciadv.1600772)
Supplement: http://advances.sciencemag.org/cgi/content/full/2/8/e1600772/DC1 [file 1600772_SM.pdf]

Science Advances

AAAS

advances.sciencemag.org/cgi/content/full/2/8/e1600772/DC1

Supplementary Materials for

A simple high-performance matrix-free biomass molten carbonate fuel cell without CO_2 recirculation

Rong Lan and Shanwen Tao

Published 17 August 2016, *Sci. Adv.* **2**, e1600772 (2016)
DOI: 10.1126/sciadv.1600772

This PDF file includes:

- fig. S1. The SEM pictures of charcoal and wood.
- fig. S2. EDS spectra of charcoal and wood.
- fig. S3. The OCV of the charcoal (□) and wood (○) fuel cell.
- fig. S4. TG-DSC analyses of charcoal and wood.

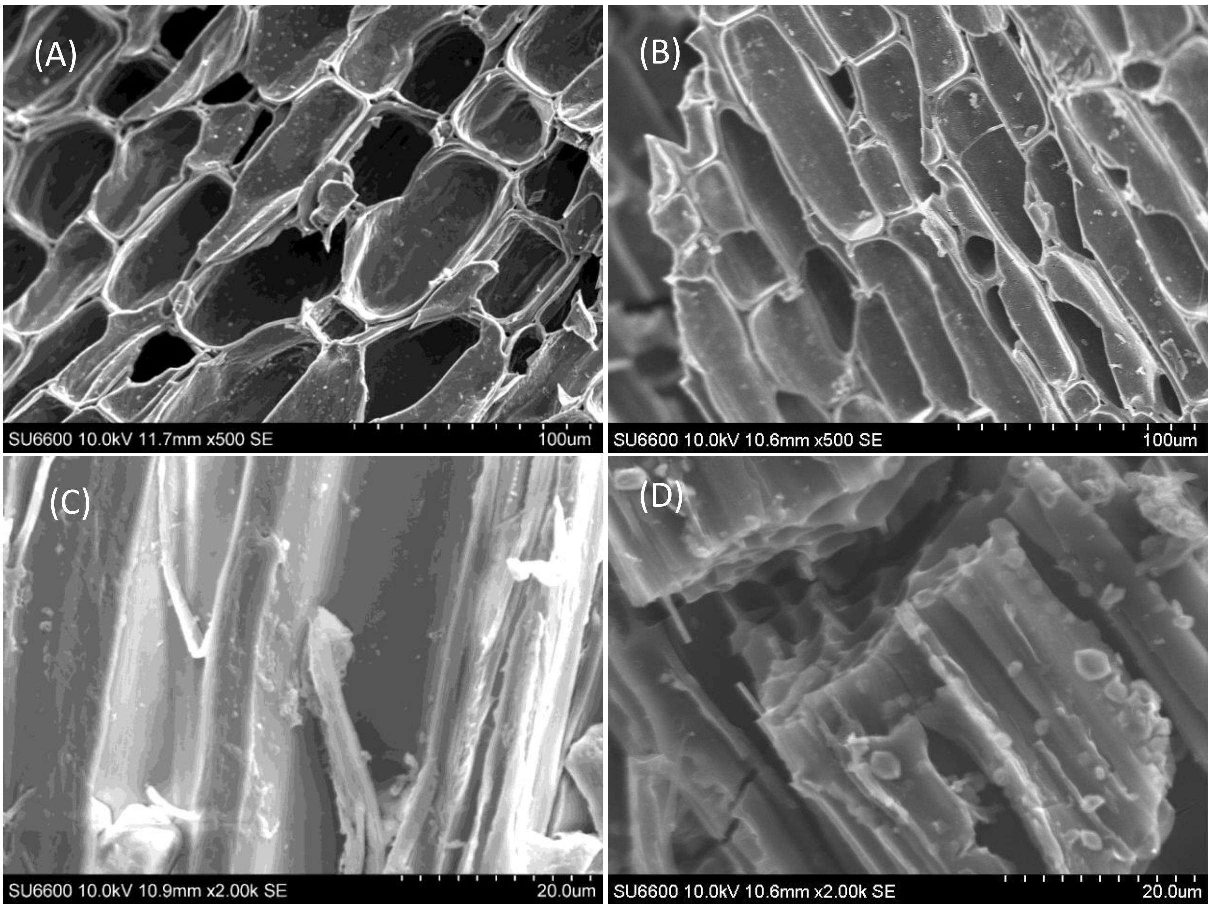

fig. S1. The SEM picture of the charcoal before (**A**) and after (**B**) and, the wood before (**C**) and after (**D**) fuel cell measurements.

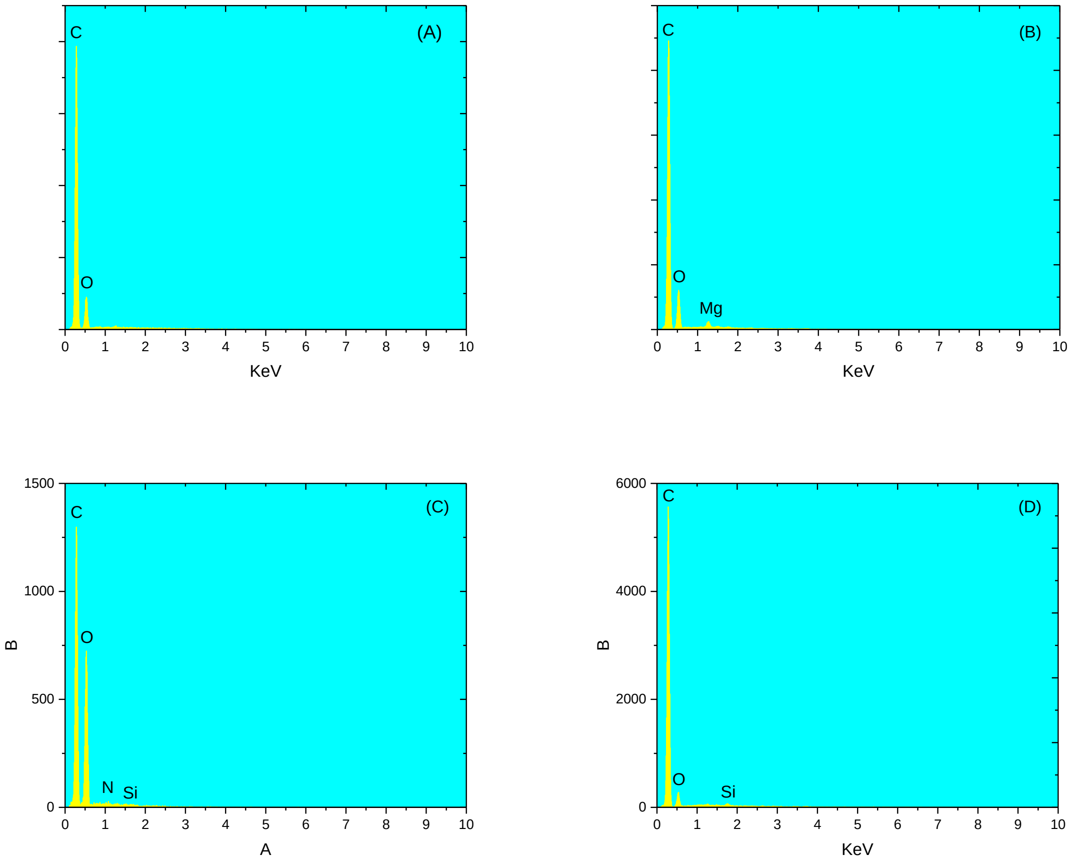

fig. S2. The typical EDS spectra of the charcoal before (**A**) and after (**B**) and, the wood before (**C**) and after (**D**) fuel cell measurements.

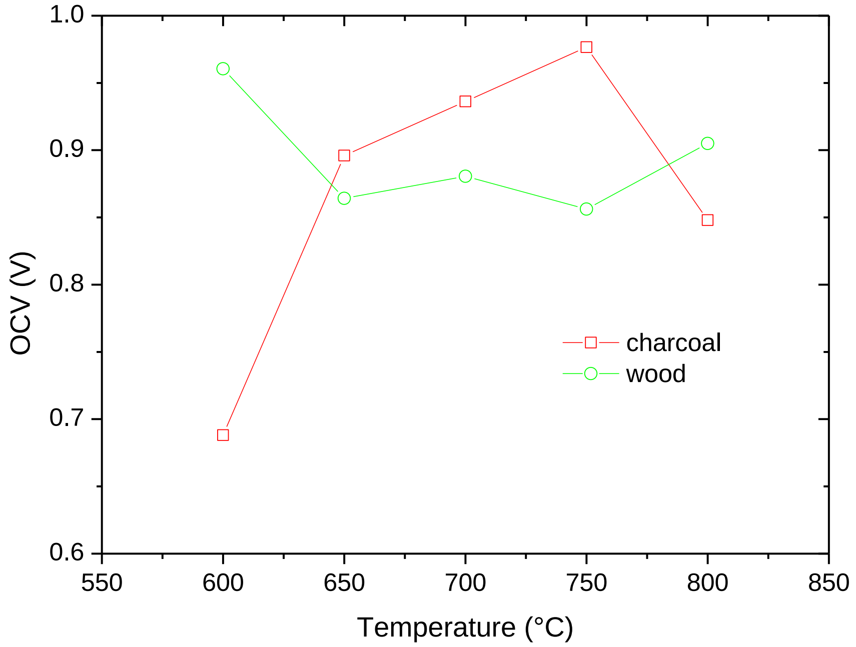

fig. S3. The open circuit voltage of the charcoal (☐) and wood (○) fuel cell.

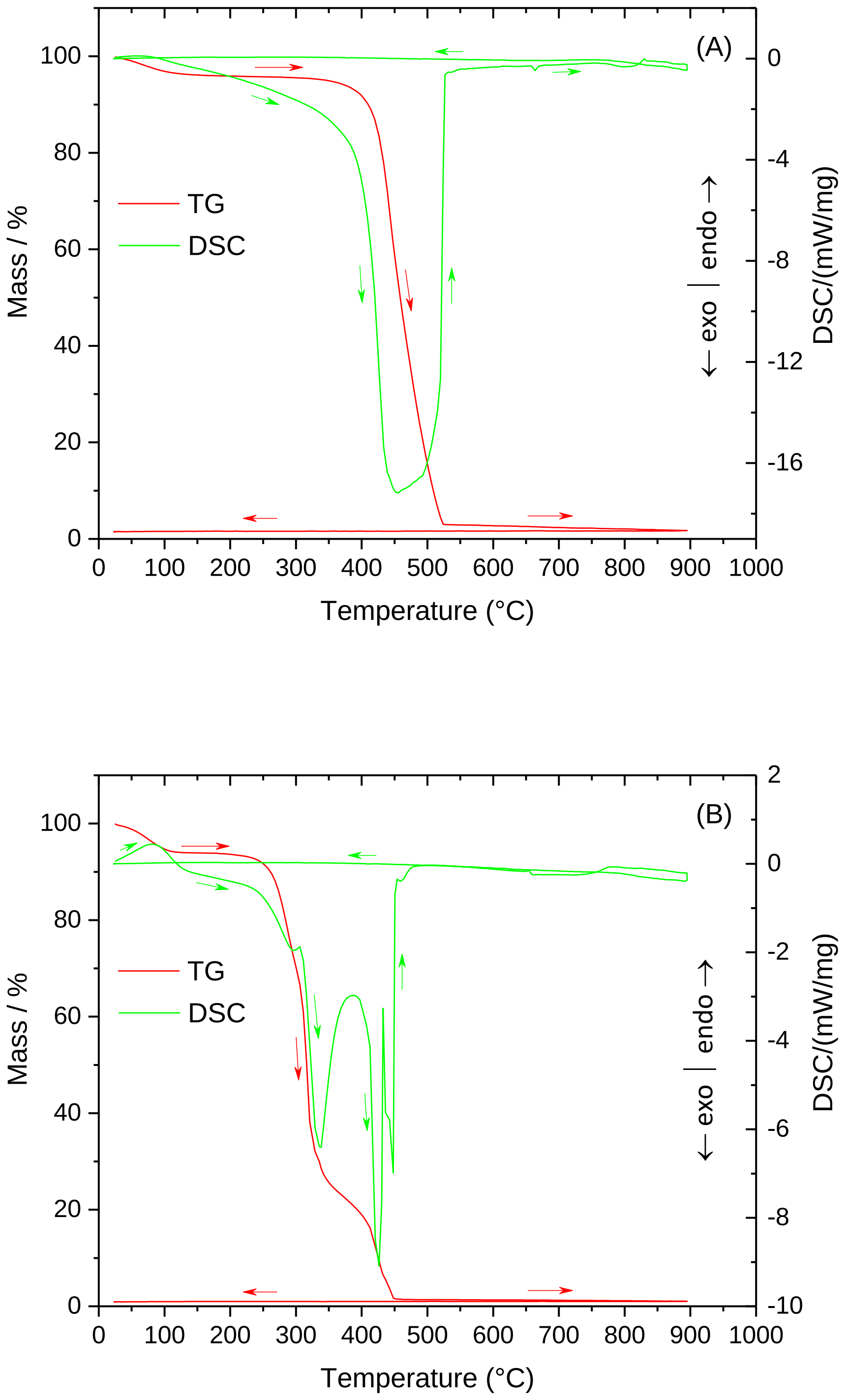

fig. S4. TG-DSC analyses of charcoal (**A**) and wood (**B**) in air between room temperature and 900°C.
